# Antibody profiles to plasmodium merozoite surface protein-1 in Cambodian adults during an active surveillance cohort with nested treatment study

**DOI:** 10.1186/s12936-015-1058-8

**Published:** 2016-01-08

**Authors:** Michele D. Spring, Sathit Pichyangkul, Chanthap Lon, Panita Gosi, Kosol Yongvanichit, Utaiwan Srichairatanakul, Amporn Limsalakpeth, Chaiyaporn Chaisatit, Soklyda Chann, Sabaithip Sriwichai, Montida Auayapon, Suwanna Chaorattanakawee, Sheetij Dutta, Satharath Prom, Char Meng Chour, Douglas S. Walsh, Evelina Angov, David L. Saunders

**Affiliations:** Armed Forces Research Institute of Medical Sciences (AFRIMS), Bangkok, Thailand; Center of Excellence for Biomedical and Public Health Informatics (BIOPHICS), Bangkok, Thailand; Walter Reed Army Institute of Research, Silver Spring, MD USA; Royal Cambodian Armed Forces, Phnom Penh, Cambodia; National Center for Parasitology, Entomology and Malaria Control (CNM), Phnom Penh, Cambodia; SUNY-Upstate Medical University, Syracuse, NY USA

**Keywords:** Malaria, Antibodies, Merozoite surface protein 1, Biomarker, Chemoprophylaxis

## Abstract

**Background:**

In addition to evidence for a protective role of antibodies to the malaria blood stage antigen merozoite surface protein 1 (MSP1), MSP1 antibodies are also considered as a marker of past malaria exposure in sero-epidemiological studies.

**Methods:**

In order to better assess the potential use of MSP1 serology in malaria chemoprophylaxis trials in endemic areas, an analysis for the prevalence of antibodies to both *Plasmodium falciparum* and *Plasmodium vivax* MSP1_42_ in healthy Cambodian adults was conducted at two sites as part of an active, observational cohort evaluating the efficacy of dihydroartemisinin-piperaquine (DP) for uncomplicated malaria (ClinicalTrials.gov identifier NCT01280162).

**Results:**

Rates of baseline sero-positivity were high (59 and 73 % for PfMSP1_42_ and PvMSP1_42_, respectively), and titers higher in those who lived in a higher transmission area, although there was little correlation in titers between the two species. Those volunteers who subsequently went on to develop malaria had higher baseline MSP1_42_ titers than those who did not for both species. Titers to both antigens remained largely stable over the course of the 4–6 month study, except in those infected with *P. falciparum* who had multiple recurrences.

**Conclusion:**

These findings illuminate the difficulties in using MSP1_42_ serology as either a screening criterion and/or biomarker of exposure in chemoprophylaxis studies. Further work remains to identify useful markers of malarial infection and/or immunity.

## Background

As US armed forces continue to deploy to malaria endemic areas for both military and humanitarian missions, development of efficacious anti-malarial chemoprophylaxis with convenient regimens and minimal side effects remains a top priority for the Department of Defense [1]. Weekly mefloquine administration is associated with well-described neuropsychiatric toxicity requiring mental health evaluation, and recently received a 2nd black box warning for this from the US Food and Drug Administration (FDA) [2, 3]. Currently favoured options include daily doxycycline, which may have lower compliance than mefloquine and considerable side effects, or atovaquone/proguanil, which is both expensive and susceptible to resistance [4].

Historically, chemoprophylaxis agents have been licensed based on the results of placebo-controlled studies in semi-immune volunteers. However, this approach raises two ethical dilemmas—justification for the use of placebo and post-trial access for the study population [5]. Non-inferiority studies between active comparator drugs is an alternative approach hampered by the logistical challenges and costs associated with very large sample sizes needed to compare products with 95 % or better efficacy. This was underscored in a trial comparing weekly mefloquine with tafenoquine in Australian soldiers deployed to East Timor. While there were no cases of *Plasmodium falciparum* malaria in either treatment arm, true efficacy could not be determined due to lack of a surrogate endpoint for malaria exposure [6], and required an estimate based on attack rates in nearby indigenous personnel [7]. Another key challenge is extrapolation of chemoprophylactic efficacy results from semi-immune populations living in endemic areas to non-immune travelers, a population who may be at great risk for more severe illness.

A surrogate biomarker for malaria exposure would ensure future active comparator trials are interpretable. Antibodies to blood stage malaria antigens, such a merozoite surface protein 1 (MSP1), have been evaluated in sero-epidemiological surveys as estimates of malaria exposure [8, 9]. The serologic stability of MSP1 make it an attractive candidate as a surrogate endpoint of exposure for chemoprophylaxis trials, but unfortunately, in a proof-of-concept *P. falciparum* controlled human malaria infection (CHMI) study by Moon et al. [10], antibodies to PfMSP1_42_ were not induced in malaria-naive, healthy volunteers taking mefloquine chemoprophylaxis with strict clinical and parasitologic monitoring; however, it may be possible that assessment MSP1_42_ as a surrogate biomarker in endemic populations may be of more utility.

The US and Cambodian militaries have recently been working to develop new anti-malarial chemoprophylaxis agents [11]. Malaria in Cambodia is characterized by a low incidence of *P. falciparum* and *Plasmodium vivax* infections in roughly equal proportion, focal transmission by forest-dwelling *Anopheles* mosquitoes with sporadic infections, all occurring in the epicenter of anti-malarial resistance [12–15]. Cambodian military personnel deploying from the non-endemic urban areas of Cambodia to forested areas along the border may be essentially malaria-naïve and at risk. This investigation assessed MSP1_42_ titers in a cohort of healthy asymptomatic Cambodian soldiers in a malaria endemic area [16] in order to evaluate its utility as a biomarker for pre-existing immunity as well as a surrogate endpoint of malaria exposure for future chemoprophylaxis studies.

## Methods

### Study design

Serum samples were isolated from 5 ml of peripheral blood drawn from volunteers enrolled in an IRB-approved study conducted in Anlong Veng District, Oddor Meanchey Province, Cambodia. From September 2010 until February 2011, a two-arm, randomized, open-label trial of 2- versus 3-day treatment regimen of dihydroartemisin-piperaquine (DP) nested within an active observational cohort study was conducted as previously reported (ClinicalTrials.gov identifier NCT01280162) [16]. Briefly, 256 volunteers were recruited and screened from two sites in Anlong Veng District, designated Site A and Site B. The former was near a village along the forest fringe, while the latter was in a remote, densely forested area. Two hundred twenty-two volunteers were enrolled and followed until 80 volunteers (representing a 40 % cumulative attack rate over 4 months) became infected with uncomplicated *P. falcipa*rum or *P. vivax* malaria and were treated with 2 or 3 days of DP. Blood was drawn for antibody titers at screening/enrollment, time of malaria infection, 42 days after completion of DP therapy, and any time of malaria recurrence. Recurrent cases were distinguished as recrudescence or reinfection by genotyping for MSP-1, MSP-2, and GLURP (glutamate-rich protein) allelic variants as previously described [17]. Genotyping for *P. vivax* recurrences was not performed.

### Antigens

The 3D7 allele of the 42-kDa recombinant protein, *P. falciparum* merozoite surface protein-1 (MSP-1_42_), was provided by Dr. Evelina Angov, manufactured at WRAIR as described in [18]. This is an *E. coli* expressed, recombinant protein with 391 amino acids, of which 17 are non-MSP-1_42_ amino acids fused to the N-terminus (bp 4087–5208, GenBank accession number Z35327, encoding amino acids 1362–1736).

*Plasmodium vivax* merozoite surface protein-1 (MSP-1_42_), was provided by Dr. Sheetij Dutta, WRAIR, and is also an *E. coli* expressed, recombinant protein based on the *P. vivax* Sal I allele, 380 amino acids long, consisting of 18 non-MSP-1_42_ amino acids fused to the N-terminus part of MSP-1_42_ (amino acids 1350–1789) [19]. Also assessed in preliminary enzyme-linked immunosorbent assay (ELISA) comparisons of titers were the CAMP and FVO alleles of *P. falciparum* MSP-1_42_, kindly provided by Dr. Evelina Angov.

### ELISA methods

Plates were coated with 100 µl PfMSP1_42_ and PvMSP1_42_ at 1 µg/ml and allowed to incubate overnight at 4 °C. Plates were washed four times and blocked with 3 % bovine serum albumin-phosphate buffered saline with Tween 20 (BSA-PBST) for 1 h; the samples were loaded at serial twofold dilution. After 2 h incubation at 37 °C, plates were washed four times and incubated for 2 h at 37 °C with peroxidase-conjugated anti-human IgG(γ) (KPL, Inc., Gaithersburg, MD, USA) at 1:8000. The plates were washed four times and incubated with ABTS substrate (KPL, Inc., Gaithersburg, MD, USA) for 30 min at room temperature. The reaction was stopped with stop solution (KPL, Inc., Gaithersburg, MD, USA) and plates read on an automatic plate reader Spectromax340PC. The absorbance at 405 nm was determined for each well with the resulting data applied to a four parameter logistic curve using Softmax Pro Version 5.2 software (Molecular Devices Corporation, CA, USA). The serum dilution required to yield an optical density of 1.0 was defined as the titer. Positive controls consisted of plasma samples from highly reactive sera. Negative controls consisted of pooled plasma from individuals from Thailand obtained through the Thai Red Cross. Before running all study samples, 10 random serum samples were selected to compare ELISA titers among the three main haplotypes of PfMSP1_42_ (CAMP, FVO and 3D7). The titers were similar among the three alleles (data not shown), suggesting strong serologic cross-reactivity in this small sample subset from Cambodia. Thus the Pf3D7 allele was selected for all study ELISAs.

### MSP1 42-kDa region genotyping (PCR and sequencing)

Genotyping of PfMSP1 42-kDa region was determined by using semi-nested PCR, and PfMSP1_19_ then sequenced to determine allelic haplotype. Primer sequences (Prima Scientific Co., Ltd.,) were as described previously [20]. The PCR products from the primary PCR, 1124 and 1072 bp for MAD20 or K1, respectively, were used as template for amplifying PfMSP1_19_ which yielded 435 and 426 bp of PCR product, respectively. Genomic DNA was extracted by using MagNA Pure compact instrument (Roche Diagnostics Ltd., Rotkreuz, Switzerland) according to manufacturer’s instruction. The primary and secondary PCR reactions were carried out in 25 µl reaction volume on Peltier Thermal Cycler (MJ Research, Waltham, MA, USA). Master Mix of both PCRs was comprised of 1× PCR buffer with MgCl_2_ (Qiagen^®^, Valencia, CA, USA), 0.1 µM of primers, 0.2 mM of dNTP (Roche Applied Science, Germany) 0.1 U/µl of Taq polymerase (Qiagen^®^ Valencia, CA, USA), and extracted DNA (adjusted concentration to 3 ng/µl). PCR conditions were as follows: pre-denaturation at 94 °C for 1 min followed by 40 cycles (35 cycles for secondary PCR) of denaturation at 94 °C for 45 s, annealing at 53 °C for 45 s (55 °C for secondary PCR), extension at 60 °C for 5 min. The PCR products were loaded on 1 % agarose gel, stained with ethidium bromide, and visualized under UV illumination (VersaDoc^®^, BIORAD, Hercules, CA, USA). For the secondary PCR, PfMSP1_19_ variants; E/TSR/L, E/TSG/L, E/KNG/F, and Q/KNG/L with GenBank accession number HM569746, HM569747, HM569748, and HM569750, respectively, were used as reference strains in sequence alignment. PCR products were purified by using a PCR purification kit (Qiagen^®^, Valencia, CA, USA) prior to sequencing. Sequencing was performed at AIT Biotech, Pte, Ltd., (Singapore) and was analysed and aligned by using BioEdit Sequence Alignment Editor Software (version 7.2.5).

### Statistical analysis

Prism v 6.0 was used for statistical analyses. The geometric mean antibody titer at each time point was determined with 95 % confidence intervals. Negative controls were determined by ELISA analysis of serum samples from ten non-malaria infected healthy Thai adults living in Bangkok. The geomean titer of negative controls for *P. falciparum* MSP1 was 225 units and for *P.vivax* MSP1, 379 units. Cut-off for seropositivity was defined as >3 standard deviations (SDs) above the geomean, or 360 and 494 units for *P. falciparum* and *P. vivax*, respectively. Comparison of numbers of volunteers seropositive or seronegative was performed by using Fishers exact tests with two-tailed *p* values. To compare level of titers, the data was log transformed to obtain normal distribution and the geomean titers reported. Titers were compared either using unpaired *t* tests or one-way ANOVA with Tukeys post-test comparisons and comparisons between vivax and falciparum titers done using Pearson’s correlation coefficient. The multiple regression analysis was conducted separately using SAS v 9.2.

## Results

### Titers and sero-positivity to *Plasmodium falciparum* and *Plasmodium vivax* MSP1_42_ in adults in northern Cambodia

**Fig. 1:**
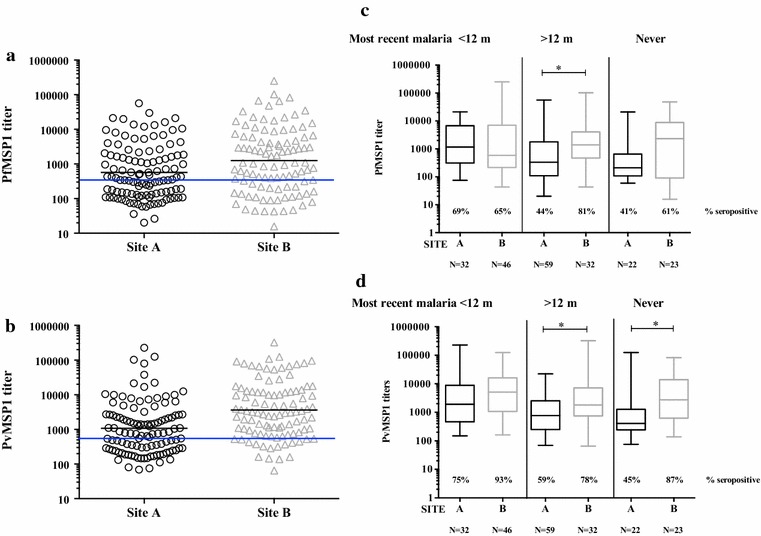
Titers to *P. falciparum* and *P. vivax* MSP1_42_ by site and clinical history. Antibody titers to *P. falciparum* MSP1_42_ (**a**, **c**) and Pv MSP1_42_ (**b**, **d**) in smear-negative volunteers at screening. **a**, **b** Titers grouped according to site: site A (n = 113) versus site B (n = 101) while **c**, **d** show titers at each site according to time since most recent malaria infection (any species): within past year, more than 1 year ago and never. **c**, **d** Also include the percent of seropositive volunteers in each timing category and site. *Black line* is geomean titer for each site, while the *blue line* represents cutoff titer for seropositivity

A total of 256 adults were screened for the study with serum samples obtained from all but one. Geometric mean titers and (range) for PfMSP1_42_ were 904 units (16–669,774) and for PvMSP1_42_ were 2207 units (65–965,671). Malaria transmission in Cambodia is seasonal, with transmission increasing during the rainy season from May to October, with peaks in September and November [21]. The majority of malaria negative volunteers, 84 % (179 of 214), were screened and enrolled during the first month of the study in September through October with a second enrollment period starting in late November to replace drop-outs [35 volunteers (16 %)]. There were 41 volunteers who were found to have malaria at screening and began treatment immediately. Of these, all nine patients with *P. falciparum* or mixed *P. falciparum*/*P. vivax* malaria were seropositive for PfMSP1_42_ with a geomean titer of 12,300 units, nearly 15× higher than those who did not have *P. falciparum* (822 units, unpaired *t* test, *p* < 0.0001). There were 32 of 41 patients with *P. vivax* malaria, and 88 % were seropositive for PvMSP1_42_, with geomean titer 4242 units, roughly 2× higher than those without *P. vivax* (2010 units, unpaired *t* test, *p* = 0.038). Among the 214 volunteers negative for malaria at enrollment, preexisting exposure based on serology was uneven at the two study sites—Site A, neighboring a small village and Site B, in a forested area (Fig. 1a). Site B had higher geomean titers for PfMSP1_42_ (1258 vs 571 units; unpaired *t* test, *p* = 0.0037) and mean PvMSP1_42_ titers of 3637 versus 1076 units, respectively (unpaired *t* test, *p* < 0.0001).

When timing of malaria episodes was grouped into <12 months ago, more than 12 months ago and never (Fig. 1b), *P. falciparum* and *P. vivax* MSP1 titers in site A were significantly higher in those with malaria within 12 months than for those not having malaria within the past year or never, *p* = 0.0117 and *p* = 0.0057, respectively (one-way ANOVA with Tukeys post-test). At site B, titers to both antigens were not different among the three groupings of malaria timings: PfMSP1 (*p* = 0.7734) and PvMSP1 (*p* = 0.1777, one-way ANOVA).

For malaria negative volunteers at enrollment, Site B participants were more often seropositive to PfMSP1_42_: 70/101 or 69 %, compared to Site A participants: 57/113 or 50 % (*p* = 0.0036 Fishers exact test). The same was true for PvMSP1_42_ with 88/101 (87 %) seropositive at site B versus 69/113 (61 %) at site A (*p* < 0.0001, Fishers exact test). Seropositivity rates per last reported malaria episode (three groupings) are shown in Fig. 1b underneath titer ranges. At Site A, there was a trend in decreasing number of seropositive volunteers with increasing time since last infection, while at Site B there was little difference in rates of seropositivity and last recalled malaria episode. Despite giving a history of never having malaria, 40 and 45 % of volunteers from Site A were seropositive for PfMSP1_42_ and PvMSP1_42_, respectively, with even higher rates for Site B: 61 and 87 %. Titers for both MSP1_42_ antigens were similar if the volunteer reported having malaria 1, 2, 3, or >3 times in the past year (PfMSP1_42_ titers 840, 1960, 4526 and 852 units respectively, one-way ANOVA, *p* = 0.13 and PvMSP1_42_ titers 3912, 3157, 2108, 6437 units respectively, one way ANOVA, *p* = 0.65). Although nearly half (49 %) of volunteers were seropositive for both *P. falciparum* and *P. vivax* MSP1 at enrollment and only 17 % seronegative for both, there was only a weak correlation of titer levels between falciparum and vivax MSP1_42_ at site A (r = 0.3262, Pearson correlation coefficient, *p* = 0.0004), and no correlation at site B (r = 0.123, Pearson correlation coefficient *p* = 0.2216).

### Titers at enrollment and subsequent malaria infection

**Fig. 2:**
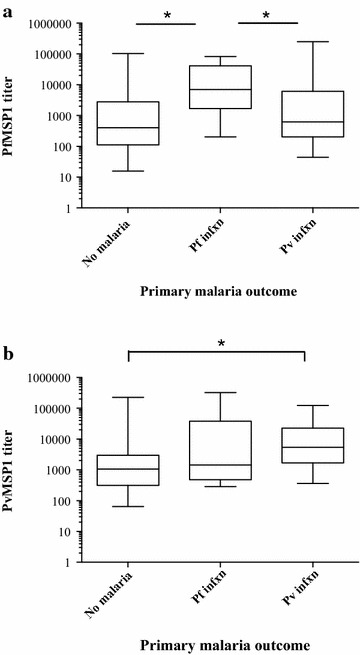
MSP1_42_ titers at enrollment and malaria outcome. Antibody titers to *P. falciparum* MSP1_42_ (**a**) and Pv MSP1_42_ (**b**) in smear-negative volunteers at enrollment based on the species of the first malaria infection contracted during the study or if remaining malaria negative. For PfMSP1, among three groups one-way ANOVA *p* = 0.0012, with Tukey’s post-test significant for *P. falciparum* infection compared to both *P. vivax* infection and no malaria. PvMSP1 titers among three groups: one-way ANOVA *p* < 0.0001 with Tukey’s post-test only significantly different between *P. vivax* titers and no malaria

Among181 malaria-negative volunteers enrolled, 50 subsequently developed malaria within 8–114 days after enrollment: 37 *P. vivax*, 10 *P. falciparum*, two mixed *P. falciparum*/*P. vivax*, and one *Plasmodium malariae* cases. *P. falciparum* and *P. vivax* MSP1_42_ titers of 49 were assessed to evaluate the hypothesis that those with higher titers at enrollment would be protected against subsequent infection with malaria, possibly confounding a chemoprophylaxis study conducted in an endemic area. For both antigens, titers at enrollment were highest in those who developed subsequent homologous infection (Fig. 2a, b). PfMSP1_42_ titers at enrollment in those subsequently contracting *P. falciparum* malaria were 6445 units, more than 10× higher than those who never developed malaria, 608 units (n = 126), and higher than those who developed *P. vivax* infection, 993 units (n = 37) (one-way ANOVA, *p* = 0.0002, with Tukeys post-test). Similarly, the titers to PvMSP1_42_ at enrollment were higher for those who subsequently contracted *P. vivax* malaria (6105 units) than those who never developed malaria (1158 units), or *P. falciparum* malaria (3235 units) (one-way ANOVA *p* < 0.0001, Tukeys post-test).

When analysed using multiple regression model, adjusting for time to infection and site of enrollment, those with higher titers to PfMSP1_42_ at enrollment were still more likely to develop subsequent *P. falciparum* malaria than those who remained malaria-free over the course of the study (*p* = 0.0065). Likewise, volunteers subsequently developing *P. vivax* malaria had higher baseline PvMSP1_42_ titers than those remaining malaria-free (*p* = 0.0006). This positive association also held for seropositivity. Of the 74 PfMSP1_42_ volunteers seropositive at enrollment, 12 % went on to develop *P. falciparum* malaria compared to 2 % of 62 seronegative volunteers (relative risk 7.54, 95 % CI 0.98–57.9) while 39 % percent of those PvMSP1_42_ seropositive subsequently developed *P. vivax* malaria versus 1 % of those who were PvMSP1_42_ seronegative (relative risk 27.1, 95 % CI 3.8–193).

Since the ELISA assay plate antigen was the 3D7 allele of PfMSP1_42_, the relationship between 3D7 antibody titers at enrollment and the infecting *P. falciparum* 42-kDa haplotype was examined. Protection by 3D7 MSP1_42_ antibodies against homologous *P. falciparum* 3D7 infection could not be determined since sequencing of the 33 *P. falciparum* isolates revealed no 3D7/MAD20 alleles. The majority of isolates (26/33 or 79 %) were the 42-kDa CAMP allele, comprised of the MAD20 33-kDa plus the EKNGL 19-kDa haplotypes, while a minority (21 %) bore the FVO allele, comprised of the K1 33-kDa plus the QKNGL 19-kDa haplotype [20, 22]. Of those who had two *P. falciparum* infections, only one volunteer (178) had a new second *P. falciparum* infection (versus recrudescence for others). This volunteer had an initial CAMP infection in October followed by FVO infection in January at the time of discharge. The *P. falciparum* MSP1 3D7 titers rose from 6120 to 293,896 units, the third highest measured of any volunteer, suggesting recent exposure and induction of immune response toward the new parasite, albeit non-protective.

### Do multiple malaria recurrences influence MSP1 titers?

**Fig. 3:**
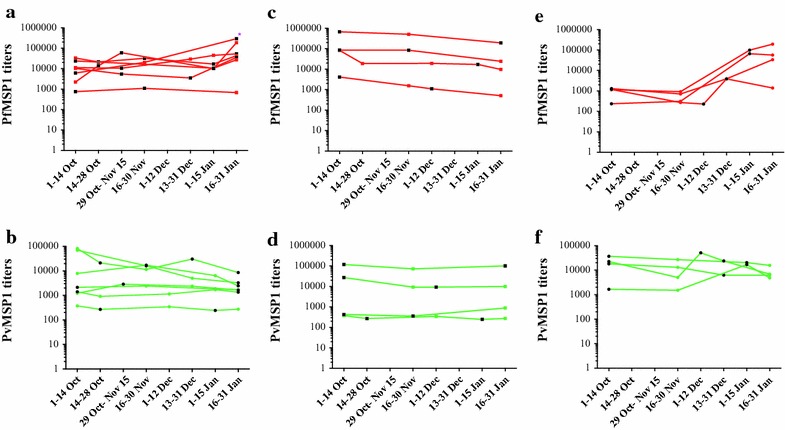
Longitudinal titers per species of infection and recurrence. **a**, **c**, **e** PfMSP titers (*red*); **b**, **d**, **f**: PvMSP1 titers (*green*). **a** Group 1 Pf titers in those seven volunteers with 2 or 3 *P. falciparum* or mixed *P. falciparum*/*P. vivax* infections; *asterisk* indicates the one volunteer with a new *P. falciparum* infection, not recrudescence. **b** Group 1 with 7 representative volunteers with *P. vivax* infections and *P. vivax* relapses only. **c**, **d** Titers in those with mono—*P. falciparum * infection followed by mono—*P.* *vivax* infection. **e**, **f** Titers in volunteers with mono—*P. vivax * infection followed by mono—*P. falciparum * infection. Study period is divided into approximately 2 week blocks along X axis. *Geometric symbols* indicate a clinic visit with serology blood draw. Those symbols in *black* are ones with a positive malaria blood smear. All diagnoses are by PCR-corrected blood smears

To look more closely at how multiple malaria infections may affect titers, the titers of volunteers with one or more recurrences are plotted and organized by groups in Fig. 3. Group 1 had at least two homologous infections, initial *P. falciparum* infection with *P. falciparum* recurrence or initial *P. vivax* infection with *P. vivax* recurrence.  Group 2 had initial *P. falciparum* infection followed by *P. vivax*, and Group 3 had initial *P. vivax* infection followed by *P. falciparum*. Titers to PfMSP1_42_ remained relatively stable over the course of the study in those with recurrent mono-infections with the exception of volunteers with initial *P. vivax* infection then *P. falciparum* infection, in which titers increased substantially with the *P. falciparum* infection. The single volunteer (178) who developed a new second *P. falciparum* infection (vs recrudescence) also had increasing titers at the new *P. falciparum* infection. Mixed infections induced varying titers although the number of volunteers was small. PvMSP1_42_ titers remained quite stable over time in all groups.

### Change in titers at study period end

**Fig. 4:**
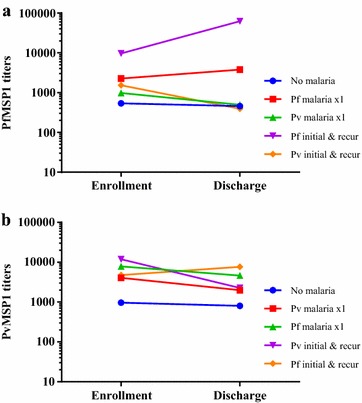
Change in titers from enrollment to discharge. Antibody titers to PfMSP1_42_ (**a**) and PvMSP1_42_ (**b**) both at enrollment and discharge grouped by no malaria infection during the study, *P. falciparum* or *P. vivax* infection one time only and those with two or more *P. falciparum* or *P. vivax* infections

In Fig. 4, titers are graphed by groups according to species of intervening malaria infection to assess if malaria infections could be detected by interval change in titers. For both PfMSP1_42_ and PvMSP1_42_, there was no statistically significant difference in titers between enrollment and discharge for any of the groups except for the four volunteers who developed multiple *P.falciparum* infections over the course of the study in whom the titers were much higher at discharge than enrollment (62,593 versus 9631 units, *p* = 0.031 unpaired *t* test). Of the 11 total *P. falciparum* infections in this group of four patients, 10 had a MSP1 19-kDa haplotype of EKNG. At discharge, titers for these subjects with multiple *P. falciparum* infections were higher than those who had never had malaria (457 units) as well as those who had one *P. vivax* infection (497 units) (*p* < 0.0001, one-way ANOVA, Tukeys post-test) but not those who had one *P. falciparum* infection (3784 units). Titers to PvMSP1_42_ did not differ significantly among the groups (*p* = 0.1328, one-way ANOVA).

## Discussion

The US military continues to develop new anti-malarial drugs and vaccines as part of its mission to protect the war fighter. Historically, anti-malarial chemoprophylaxis studies for licensure in Asia and elsewhere have used placebo-controlled study designs in semi-immune populations, with recent examples including tafenoquine in soldiers of the Royal Thai Army [22] and azithromycin in Indonesian soldiers [23]. However, the declaration of Helsinki 2000 raised serious concerns regarding placebo-controlled study designs where alternative effective therapies exist [5]. An established, validated biomarker for malaria infection would allow for calculation of protective efficacy in active-controlled malaria prevention studies. To this end, in 2009, Moon et al. [10] performed a CHMI study administering *P. falciparum* to healthy volunteers under mefloquine prophylaxis to assess seroconversion rates of *P. falciparum* MSP1_42_, defined as a fourfold rise in titers. None of the volunteers seroconverted, and only four of six controls, those who did not receiving mefloquine who did develop malaria, seroconverted.

This study aimed to characterize background and changes in humoral *P. falciparum* and *P. vivax* MSP1_42_ responses during a single malaria season in order to gain insight to malaria exposure and immunity in a low transmission setting with multidrug resistant malaria in northern Cambodia and lay the groundwork for malaria prophylaxis studies. The predominantly resident military population was a mix of self-reported malaria-naïve and semi-immune individuals; some were newly arrived from non-transmission areas while other long-term veterans had likely had multiple exposures over several years while stationed in malarious areas. Transmission varied substantially between the two study locations, with significantly higher attack rates during the cohort study at the forested site B compared to semi-urban site A [16]. Baseline titers to *P. falciparum* and *P. vivax* MSP1_42_ proteins in asymptomatic, aparasitemic adults varied greatly by individual, with a 1–5 log difference not directly attributable to geographic site nor reported clinical history of prior malaria infection. Geomean titers were higher in those reporting a malaria infection in the past year compared to those with no history of disease, but significant overlap existed, underscoring the lack of utility of a single serological measurement as marker of immunity. Based on sero-positivity rates, prior exposure seemed quite evident, particularly for *P. vivax*, even in those reporting no clinical history of malaria.

The wide variation in baseline serology is similar to prior sero-epidemiology studies in both high and low transmission settings. Fowkes et al. [24] found that titers to merozoite surface antigens *P. falciparum* and *P. vivax* AMA1 could fluctuate widely in pregnant women living in Thailand even when measured biweekly. Another study in northern Thailand found that in individuals with documented falciparum or vivax malaria infection in the past 6 years, only 48 % were sero-positive to PfMSP_19_ and 11 % to *P. vivax* MSP1_19_ [25]. Even in a hyperendemic transmission area in western Kenya, approximately 60 % of semi-immune children and adults surveyed did not have a humoral response to any of the three main allelic variants (CAMP, FVO and 3D7/MAD20) of PfMSP1_42_ [26]. This lack of correlation with reported malaria exposure history raises significant doubts regarding the utility of MSP1 antibodies as an enrolment criterion for malaria prevention studies in even low transmission areas requiring malaria-naïve volunteers. At best, aggregate seroprevalence data could be used to identify locations to conduct prevention trials a priori and/or to interpret study results after the fact.

Volunteers who did not get malaria during the course of the cohort study had essentially no change in titer. Mean PfMSP1 titers hovered just above the upper limit of seropositivity at both time points suggesting this is the natural ‘background’ titer of adults in rural Cambodia, although this would need to be validated during more than one transmission season. Ideally, sero-epidemiological studies conducted in areas with seasonal transmission would have humoral responses measured just before the onset of the transmission season. Since this study was nested in an active drug efficacy study, conducted several months after the rains had begun, baseline titer levels and rates of seropositivity may be overestimated. It is unclear if the high rate of sero-positivity signifies recent or remote infections, and it gives little indication of the number and species of prior infections, particularly for those with low titers. Regardless, the high rates did not appear to confer immunity.

Perhaps more importantly, there was little apparent ‘booster effect’ of baseline titers in response to infection, except for PfMSP1_42_ for the four volunteers with three malaria infections during the study period. The PfMSP1 19-kDa haplotype for 10 of 11 infections for these four patients was EKNG; thus the higher titers do not represent a broadening of the immune responses by infection with different circulating haplotypes. With *P. vivax* infections, titers appeared either to wane or remain remarkably stable over time. As reported elsewhere, it is likely that the majority of *P. vivax* infections represented relapses [27], and this would suggest that relapsing infection does not stimulate a significant rise in humoral response. A recent publication by Chuquiyauri et al. [28] analysed antibody responses to *P. vivax* antigens by microarray in Peruvian patients with *P. vivax* mono-infection and found that both height and breadth of responses were not different in those who were determined to have relapse versus *P. vivax* re-infection. Moreover, boosting may not be seen due to longer half-lives of MSP1 titers in low to medium transmission areas. Wipasa et al. [25] used a mixed-effects regression model analysis to estimate the half-life of MSP1 in northern Thai volunteers to be 7.6 years. These evidences, combined with the data obtained here in northern Cambodia, strongly suggest MSP1 is unlikely to be a useful quantitative serologic marker for malaria exposure during a chemoprophylaxis study.

Alternative biomarkers to other antigens, such as antibodies to the circumsporozoite protein (CSP), which is located on the surface of the sporozoite, may be less affected by chemoprophylaxis. In an intervention-treatment-vaccination (ITV) study in healthy Dutch volunteers, those taking chloroquine prophylaxis during three successive mosquito-borne *P. falciparum* infections were protected from subsequent *P. falciparum* challenge and seroconverted to CSP but not AMA1 or GLURP [29]. A study of Dutch soldiers under mefloquine prophylaxis while deployed to Zaire in 1994 showed 100 % efficacy while 11 % of soldiers had an increase in circumsporozoite antibodies, suggesting malaria exposure [30]. Less is known about seroconversion with *P. vivax* malaria, although one study of deployed Thai soldiers found almost three-quarters contracted *vivax* malaria despite 62 % being PvCSP seropositive at deployment; those who were seronegative did seroconvert with acute illness though the rise in titers was modest and fell quickly [31]. Thirty-eight percent of the Thai soldiers with documented, treated *P. vivax* malaria did not mount any CSP antibody response. There were not successive increases in CSP titers with relapses, a finding also demonstrated in Cambodian adults in this study, suggesting that a biomarker for *P. vivax* infection may be even more elusive. Whether pre-erythrocytic or erythrocytic, a single antigen may not suffice as a biomarker; thus, adding immunogenic antigens, such as MSP2 and schizont extract (SE) [32], or assessing the breadth of responses [33] plus newer bead arrays or microarrays [34, 35] may prove to be more useful, as biomarkers but these alternate approaches will need to be validated in chemoprophylaxis field studies.

In design of chemoprophylaxis studies in endemic areas, it is possible that innate immunity in semi-immune subjects may attenuate infection, thereby inflating the apparent protective efficacy of prophylactic drugs and overestimate their potential benefit to malaria-naïve individuals. The previous evidence for the protective role of *P. falciparum* and PvMSP1 in endemic areas has been mixed [36–39]. In this study, those with higher MPS1 titers to either species at enrollment were more likely to develop malaria, and the proportion of volunteers remaining malaria-free was almost evenly split between those who were seropositive and seronegative, suggesting these higher titers were more reflective of exposure than semi-immunity. No 3D7 haplotypes to the C-terminus of MSP1 were detected, the region of MSP1 to which functional antibodies are thought to act, yet titers to this allelic haplotype were highly induced, perhaps merely reflecting cross-reactive immune response between three haplotypes 3D7, CAMP, FVO [20, 36]. Cross-reactivity may not translate to cross-protection, although this may depend on haplotype, as it has been shown that vaccination with the FVO allele of PfMSP1_42_ induces a better homologous and heterologous antibody response than the 3D7 allele of MSP1_42_ [40]. In addition, a traditional ELISA may not adequately capture function, as some antibodies have “blocking” activities and thus interfere with protective responses [41].

## Conclusion

Evidence for the establishment and duration of both serologic markers and functional malaria immunity in subjects living in Southeast Asia remain limited. Compounding the complexities of assessment, unlike homologous laboratory strains used in challenge studies, malaria infections in endemic areas are frequently with heterologous strains or mixed species and responses reflecting the more complex transmission patterns such as observed in Cambodia. These factors combine to limit the current utility of serologic biomarkers of malaria immunity in malaria prevention studies. Studies to assess the development of a broader array of serologic biomarkers in controlled settings are needed.

## Abbreviations

AFRIMS: Armed Forces Research Institute of Medical Sciences
AMA1: apical membrane antigen 1
CHMI: controlled human malaria infection
CSP: circumsporozoite protein
DP: dihydroartemisinin-piperaquine
ELISA: enzyme-linked immunosorbent assay
FDA: Food and Drug Administration
MSP1: merozoite surface protein-1
PCR: polymerase chain reaction
WRAIR: Walter Reed Army Institute of Research
